# Optimization of the Technology of Suppositories with CO_2_ Extract of *Rosmarinus officinalis* L. and In Vitro Evaluation of Chamazulene Release

**DOI:** 10.3390/molecules31213999

**Published:** 2026-11-06

**Authors:** Aigul Medeshova, Perizat Orazbayeva, Aizhan Essedova, Meruyert Amantayeva, Yerbolat Tulebayev, Zhanel Raiymbekova, Sabyrova Sabira, Bekzhan Orazbayev

**Affiliations:** 1 School of Pharmacy, Karaganda Medical University, Gogol 40, Karaganda 100000, Kazakhstan; medeshova@qmu.kz (A.M.); sabyrovas@qmu.kz (S.S.); 2 Department of Chemical and Biochemical Engineering, K. Turysov Institute of Geology and Oil and Gas Engineering, Satbayev University, Almaty 050012, Kazakhstan; m.amantayeva@satbayev.university; 3 Department of Fundamental Medicine, Faculty of Medicine, M. Kozybayev North Kazakhstan University, Petropavl 150000, Kazakhstan; tulebaeverbolat@mail.ru; 4 Department of Clinical Pharmacology and Evidence Based Medicine, Karaganda Medical University, Karaganda 100000, Kazakhstan; 5 Department of Oncology and Radiation Diagnostics, Karaganda Medical University, Amanzholov 189, Karaganda 100000, Kazakhstan

**Keywords:** CO_2_ extract, manufacturing conditions, GC–FID, in vitro diffusion, formulation optimization, analytical method development

## Abstract

This study investigated manufacturing conditions for suppositories containing a CO_2_ extract of *Rosmarinus officinalis* L. herb, using a previously developed formulation, and evaluated a gas chromatography procedure with flame ionization detection (GC–FID) for quantitative marker determination. The effects of key manufacturing parameters were evaluated, and the optimal conditions for base preparation were determined as 70 °C and 120 rpm for 12 min; increasing the agitation speed to 150 rpm did not reduce the processing time. Extract concentrate preparation at 37 °C and 60 rpm required 25 min to achieve visually assessed homogeneity. At 60 rpm, prior concentrate preparation reduced the subsequent mixing time of the suppository mass from 100 to 20 min at each investigated temperature (37, 45, and 60 °C), excluding the time required to prepare the concentrate. Cooling at 8 °C for 60 min was selected on the basis of mechanical-strength measurements. Agar diffusion experiments provided a comparative assessment of the diffusion of colored extract constituents rather than a quantitative measure of chamazulene release or bioavailability. The findings identify candidate manufacturing conditions for further evaluation. Confirmation of marker identity, analytical validity, quantitative content uniformity, and long-term stability is required before reliable product standardization can be established.

## 1. Introduction

At present, one of the priority areas of pharmaceutical science is the development of effective and safe medicines based on natural plant-derived raw materials. Researchers are particularly interested in incorporating biologically active substances of plant origin into modern dosage forms capable of ensuring high therapeutic efficacy, bioavailability, and a minimal risk of side effects [1,2,3]. Among promising medicinal plants, *R. officinalis* L. attracts considerable attention; it is a source of a wide range of biologically active compounds, including phenolic acids, flavonoids, and diterpenes [4]. The combination of these substances accounts for the plant’s pronounced antioxidant, anti-inflammatory, antimicrobial, and cytoprotective properties, which determine its potential for use in medicinal preparations [5,6,7,8,9,10,11,12].

In recent years, supercritical CO_2_ extraction technology has become increasingly widespread for the production of plant extracts. This method enables the production of highly concentrated extracts while preserving heat-labile components, eliminates the use of organic solvents, and ensures a high degree of purity and reproducibility in the composition of the final product. Thanks to these advantages, CO_2_ extracts are regarded as a promising raw material for the development of modern herbal medicines.

However, despite a significant body of research into the chemical composition and pharmacological activity of medicinal rosemary, the development of standardized dosage forms based on its CO_2_ extract remains insufficiently studied. Of particular interest is the development of suppositories, which provide both local and systemic action of the active substances. The suppository dosage form facilitates the effective delivery of biologically active compounds through the mucous membranes, allows the first-pass effect through the liver to be partially avoided, ensures dosing accuracy, and helps to preserve the stability of thermolabile components. Furthermore, the local release of active substances helps to reduce the risk of systemic side effects and improve the safety of treatment.

An important aspect of the development of herbal medicines is ensuring their standardization and quality control. However, validated methods for the quantitative determination of biologically active components in dosage forms containing plant extracts are still lacking [13,14,15,16,17,18,19]. The development and validation of modern analytical methods enable the monitoring of active ingredient content, ensuring the reproducibility of the manufacturing process, the stability of the medicinal product, and its compliance with regulatory requirements [20,21,22,23,24,25].

Therefore, the development of a technology for suppositories containing a CO_2_ extract of *R. officinalis* L. herb and the establishment of an assay for the active components represent a pressing challenge for modern pharmaceutical technology [18,19]. Solving this problem will make it possible to combine the advantages of modern methods for extracting plant raw materials and effective dosage forms with regulatory requirements and quality assurance, thereby creating a scientific basis for the further introduction of the developed medicinal product into pharmaceutical practice.

We previously reported the selection of the composition and the physicochemical characterization of suppositories containing the CO_2_ extract of *R. officinalis* L. herb, together with their anti-inflammatory activity in a carrageenan-induced paw edema model in rats [4].

The aim of the study was to develop a manufacturing process for suppositories containing a CO_2_ extract of *R. officinalis* L. and to establish a method for the quantitative determination of biologically active components to ensure the standardization and quality control of the dosage form [26]. To achieve this objective, we investigated the effect of temperature, stirrer speed, and mixing time on the preparation of the base, the extract concentrate, and the suppository mass, as well as the effect of the cooling regime on the hardness of the molded suppositories; the GC–FID conditions for determining the marker compound and the in vitro release of the active components from different bases were also studied.

## 2. Results

### 2.1. Optimization of the Base Preparation Process for Suppositories with CO_2_ Extract of Rosmarinus officinalis L.

The selected formulation contained 0.50 g of *R. officinalis* L. CO_2_ extract per suppository, with a nominal unit mass of 1.50 g. Its composition is presented in Table 1.

The calculated amount of yellow beeswax required for one batch was loaded into a steam-jacketed reactor. Ground cocoa butter and half of the amount of flaxseed oil specified in the formulation were then added. The mixture was heated at 60, 70, or 80 °C while being stirred with a paddle agitator at 30, 60, 120, or 150 rpm. Base preparation was considered complete when no visible wax particles remained. The base-preparation times under the investigated conditions are presented in Table 2. At 60 °C, prolonged preparation times or failure to reach the visual endpoint within the observation period indicated incomplete incorporation of the wax into the base under the tested conditions.

As shown in Table 2, base preparation at 60 °C required prolonged mixing, and the visual endpoint was not reached within 180 min at stirrer speeds of 30 and 60 rpm. At 120 rpm, the reported preparation time decreased from 150 min at 60 °C to 12 min at 70 °C. Increasing the temperature further to 80 °C reduced the preparation time to 10 min. At 70 °C, increasing the stirrer speed from 120 to 150 rpm did not further shorten the reported preparation time.

Based on these observations, a temperature of 70 °C and a stirrer speed of 120 rpm were selected for base preparation, with a recorded preparation time of 12 min. This temperature was applied specifically to the preparation of the base before addition of the extract concentrate.

The prepared base was then transferred to a second reactor. A separately prepared concentrate containing the CO_2_ extract of *R. officinalis* L. herb, 96% ethanol, and the remaining portion of flaxseed oil was added. Polysorbate 80 (Tween 80) was added as an emulsifier. The mixture was stirred at temperatures of 37, 45, and 60 °C and stirrer speeds of 30, 60, 120, and 150 rpm until visually assessed homogeneity was achieved. This visual endpoint indicated the absence of visible heterogeneity and did not establish quantitative uniformity of marker distribution.

For comparison, under similar conditions, a suppository mass was prepared by directly dissolving the CO_2_ extract of *R. officinalis* L. herb in the base, bypassing the concentrate preparation stage. When introduced into the base without prior preparation of a concentrate, homogeneity of the mixture was achieved only after 1.5 h at high stirrer speeds and after 2–2.5 h at low speeds, which indicates that this method is inefficient. The results are shown in Table 3.

**Figure 1:**
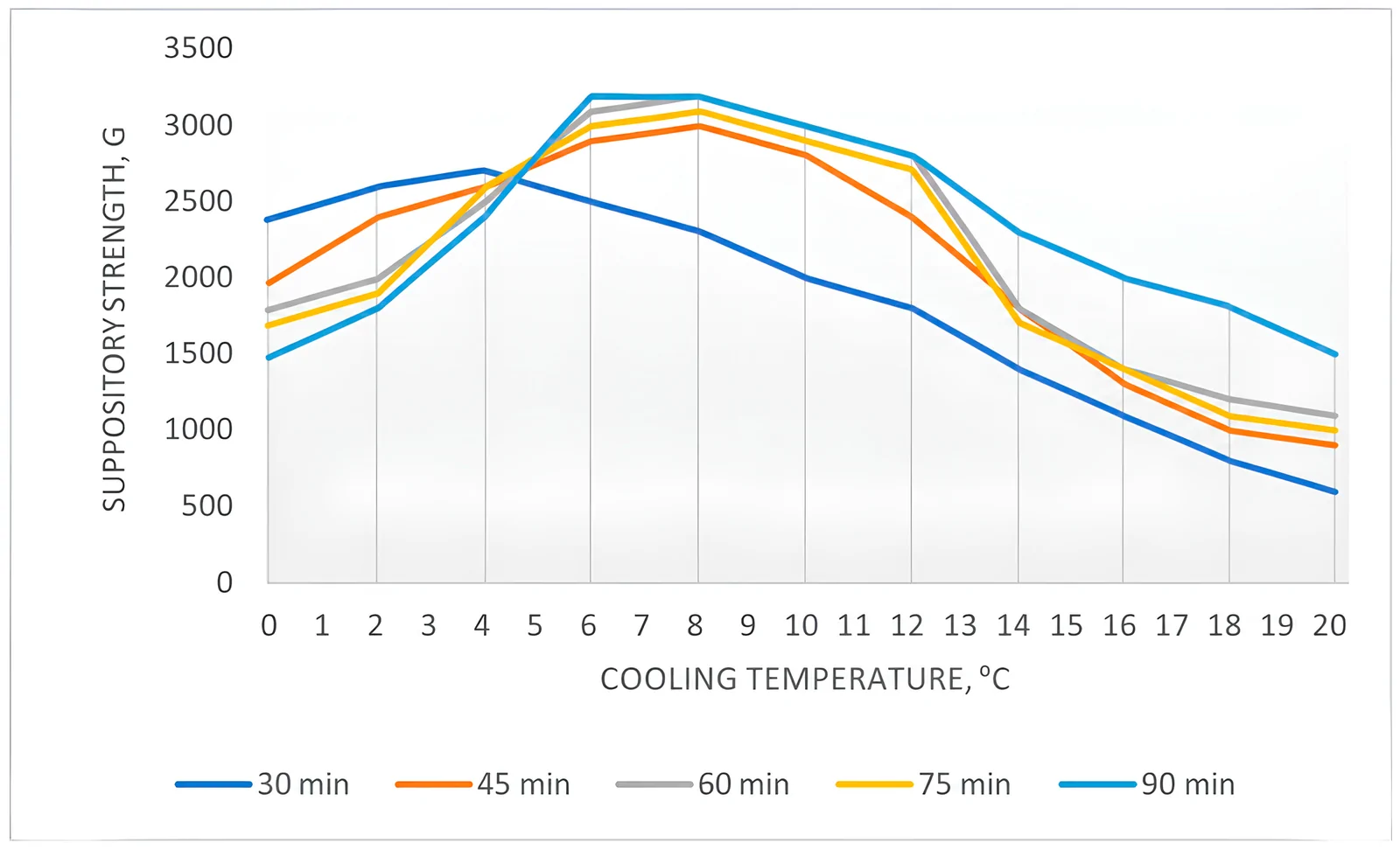
Suppository hardness as a function of cooling temperature for cooling times of 30, 45, 60, 75, and 90 min. *X*-axis: cooling temperature, °C; *Y*-axis: suppository strength, G.

In order to select the optimal process conditions during the suppository molding stage, the effect of cooling temperature and cooling time on the mechanical strength of the finished product was investigated. The suppository mass was poured into molds and cooled under the tested temperature–time regimes: 30, 45, 60, 75, and 90 min at temperatures ranging from 0 to 20 °C. For each regime, five samples were tested (n = 5). The results are presented as mean ± standard deviation (SD). The results are shown in Figure 1.

The data obtained (Figure 1) indicate that suppository strength depended on both cooling temperature and cooling time. The maximum strength was observed at 6–8 °C for all cooling durations. At 8 °C, a cooling time of 60 min provided a strength of approximately 3000 G, which was close to the maximum values obtained at 75 and 90 min. A shorter cooling time of 30 min at 8 °C yielded only about 2600 G, which was insufficient. Increasing the cooling time to 90 min did not substantially improve strength compared with 60 min. At temperatures above 12 °C, suppository strength decreased markedly, and even prolonged cooling at 20 °C did not ensure adequate hardness. Therefore, when manufacturing suppositories containing the CO_2_ extract of *R. officinalis* L., preference should be given to a longer cooling period (60 min) at a temperature of +8 °C rather than an intensive but short-term cooling process.

### 2.2. Quantitative Determination of Biologically Active Components in Suppositories Containing the CO_2_ Extract of Rosmarinus officinalis L.

The chamazulene content in suppositories containing the CO_2_ extract of *R. officinalis* L. herb was determined by GC–FID and amounted to 0.0029 g per suppository.

**Figure 2:**
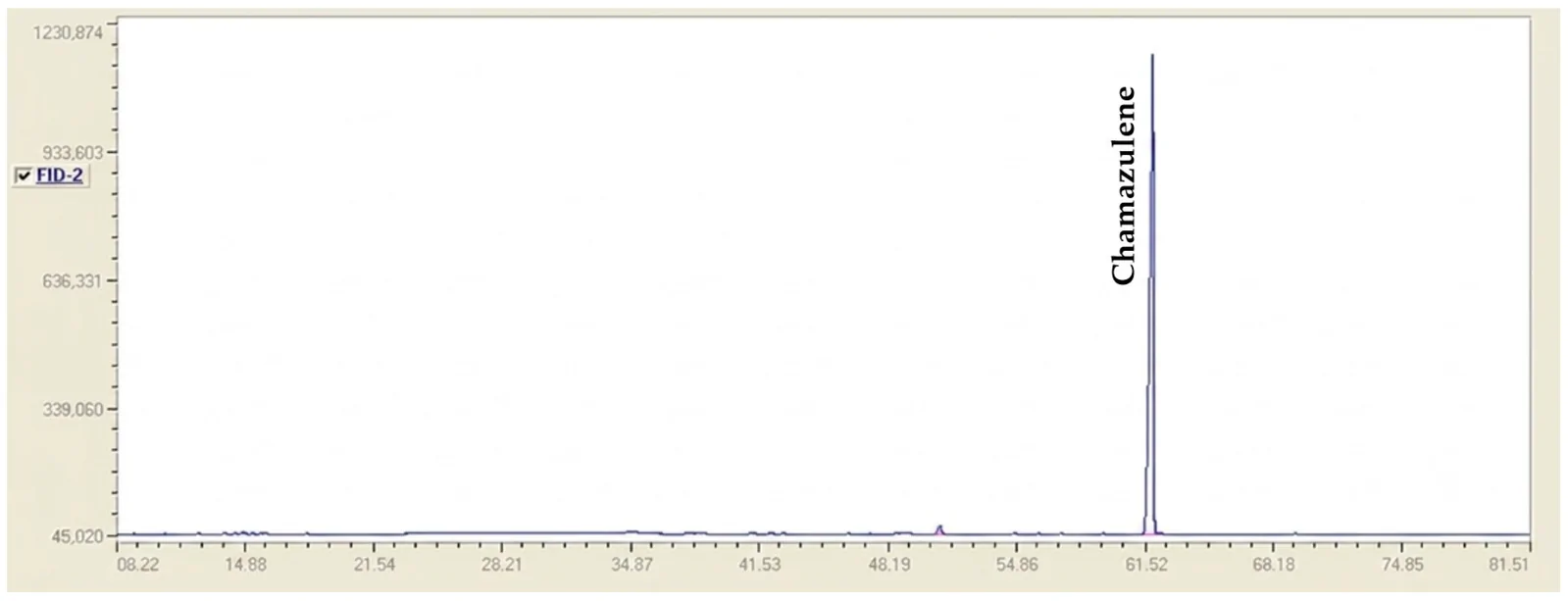
Chromatogram of the chamazulene reference standard solution.

**Figure 3:**
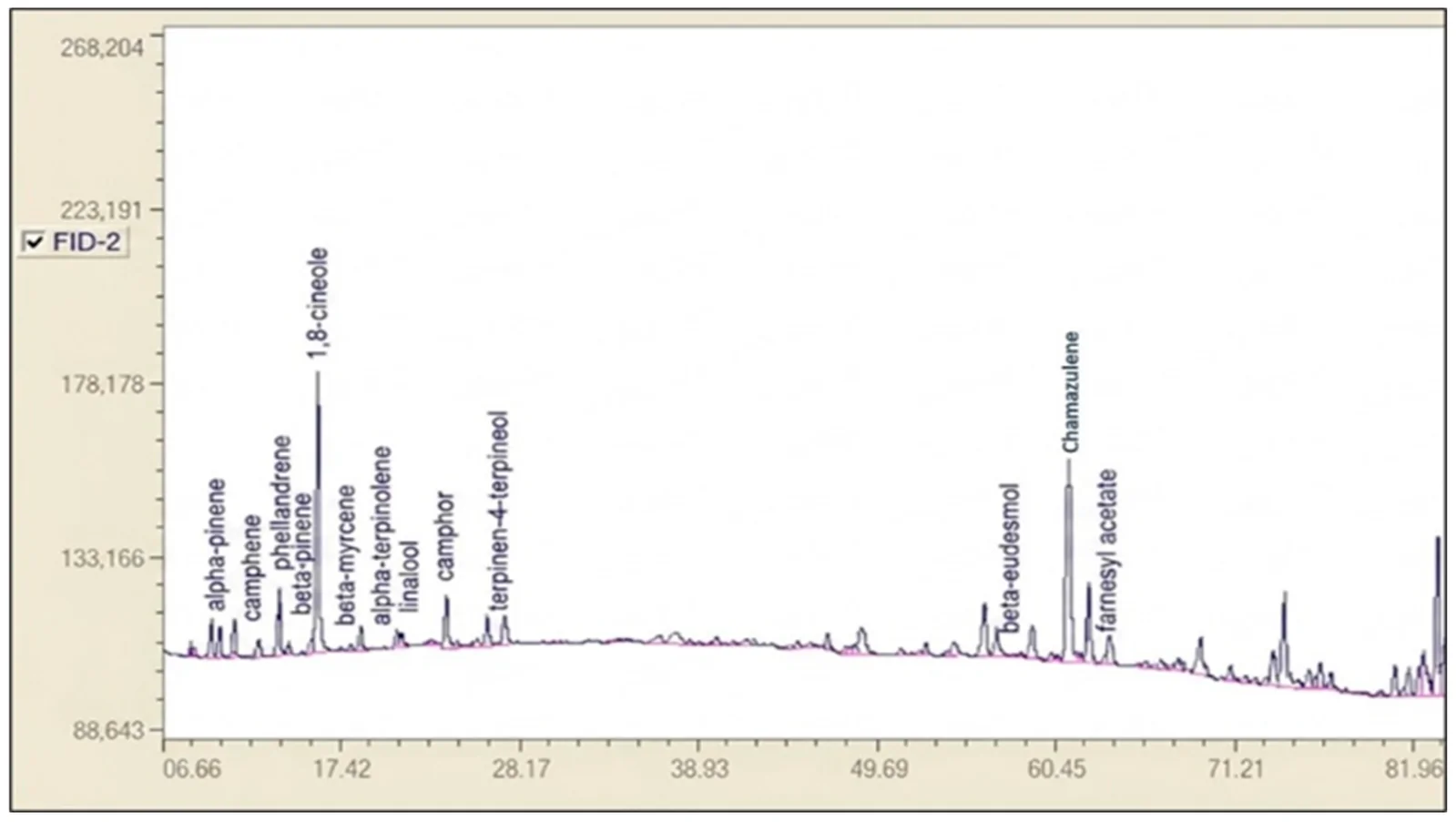
Chromatogram of suppositories containing the CO_2_ extract of *R. officinalis* L.

A standard sample of chamazulene, whose chromatographic peak is shown in Figure 2, was used to identify chamazulene in the test sample. The results of the analysis of suppositories containing the CO_2_ extract are presented in Figure 3. Chamazulene was identified by comparing the retention time of the corresponding peak in the test sample with that of the reference standard (61.52 min). The peak was additionally confirmed by spiking the test sample with the chamazulene standard. A chromatogram of the blank suppository base was used to exclude interference from the excipients. The chamazulene peak is labeled in Figure 3. These results confirm the presence of chamazulene in the suppositories under the chromatographic conditions used.

The GC–FID data also indicated a high degree of uniformity in the distribution of chamazulene throughout the suppository mass, confirming the effectiveness of the selected manufacturing conditions and gradual cooling process. The results obtained support the use of chamazulene content as a criterion for the quality control and standardization of suppositories containing the CO_2_ extract of *R. officinalis* L. herb.

### 2.3. In Vitro Diffusion of Colored Constituents from Suppositories Containing a CO_2_ Extract of Rosmarinus officinalis L.

The diffusion of extract constituents from a suppository formulation depends on their physicochemical properties, the composition of the suppository base, and the experimental conditions. To compare the behavior of the investigated formulations, an agar well diffusion assay was performed, and the diameters of the colored zones surrounding the wells were measured.

**Figure 4:**
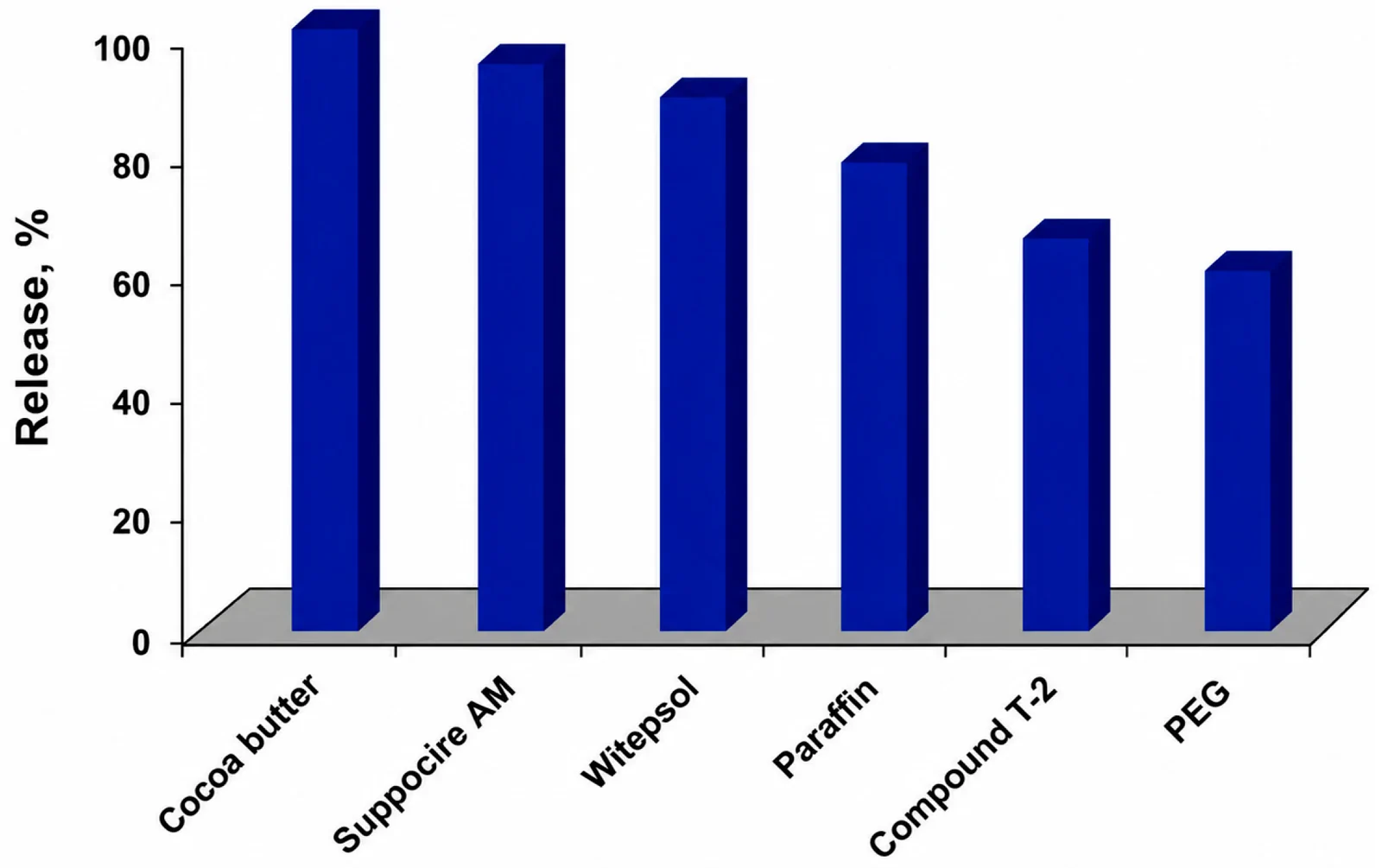
Comparative assessment of the extent of chamazulene release from suppository bases in in vitro experiments (n = 5).

The observed colored zones were attributed to the diffusion of colored constituents of the CO_2_ extract from the suppository formulations into the agar medium. The diameters of these zones were used only for comparative assessment of the formulations. Because the compounds present in the colored zones were not chemically identified or quantitatively determined in the agar medium, the results should not be interpreted as quantitative chamazulene release, release kinetics, or evidence of bioavailability. The results of the comparative diffusion assay are presented in Figure 4.

## 3. Discussion

### 3.1. Optimization of the Suppository Base Preparation Process

As part of this study, the optimization of the process conditions for the preparation of a suppository mass containing the CO_2_ extract of *R. officinalis* L. revealed that the quality of the dosage form is significantly influenced by the parameters of thermal and mechanical processing. It was established that the optimal mixing conditions—a temperature of 70 °C and a stirrer speed of 120 rpm for 12–14 min—ensure the formation of a homogeneous mass with an even distribution of biologically active components. These results are consistent with data on suppository formulations, where the optimization of mixing parameters is a key factor in ensuring process reproducibility [26,27].

The introduction of the CO_2_ extract directly into the base without prior concentration proved to be technologically ineffective: homogeneity of the mixture was achieved only after 1.5 h at a high stirrer speed and after 2–2.5 h at a low speed. This finding is consistent with the literature on the difficulty of integrating lipophilic plant extracts into suppository bases without prior preparation, which leads to prolonged mixing and the risk of degradation of the active compounds [28].

Analysis of the suppository cooling data (Figure 1) showed that preference should be given to a longer, controlled cooling period at +8 °C for 60 min, as opposed to short, intensive cooling regimes. This regime ensures the formation of a stable crystalline structure in the base and prevents phase separation of the mass, promoting the uniform distribution of the biologically active components of the CO_2_ extract. These results are supported by studies on the effect of crystallization rate on the homogeneity of suppository systems, which have shown that controlled cooling reduces internal stresses and structural defects [28]. The use of gradual cooling also has a positive effect on the stability of the extract’s thermolabile compounds. The literature indicates that volatile mono- and sesquiterpenes present in rosemary essential oil may be susceptible to volatilization, oxidation, and compositional changes under unfavorable processing conditions. Therefore, controlled cooling may help limit changes in the terpene fraction of the CO_2_ extract. However, the stability of individual terpene compounds during cooling was not directly evaluated in the present study [28].

Gradual cooling at 8 °C for 60 min ensured the formation of suppositories with higher mechanical strength compared with intensive short-term cooling. Based on the results obtained, this regime was selected as optimal for the molding stage of suppositories containing the CO_2_ extract of *R. officinalis* L. herb.

Consequently, the set of optimized parameters—preparation temperature, frequency of mechanical agitation, and cooling regime—ensures the technological stability, uniformity, and reproducibility of the suppository mass containing the CO_2_ extract of *R. officinalis* L. The results obtained not only confirm previously published findings on the importance of controlling process operations when developing suppository dosage forms but also expand our understanding of the use of CO_2_ extracts in pharmaceutical delivery systems [29,30,31,32,33,34,35,36,37,38].

Thus, adherence to the proposed preparation and cooling regimes is critical for ensuring the quality, stability, and pharmacological efficacy of suppositories containing plant extracts, which provides an objective basis for their further standardization and implementation in practical pharmaceutical practice.

### 3.2. Quantitative Determination of Biologically Active Components

Analysis of chamazulene content using GC–FID showed that its concentration in suppositories containing the CO_2_ extract of *R. officinalis* L. herb is 0.0029 g per unit of the preparation. This result confirms the effectiveness of the selected manufacturing process and the gradual cooling of the suppository mass, which helps to preserve the thermolabile bioactive compounds in the extract. The use of controlled processing conditions may help preserve thermolabile terpenoid constituents, including chamazulene, that may contribute to the biological activity of the suppository formulation [32].

The detection of chamazulene in the prepared dosage form suggests that the selected manufacturing conditions may be suitable for its incorporation into suppositories. Chamazulene content may be considered a potential quality-control parameter for this formulation. Further comparative and pharmacological studies are required to evaluate the influence of the manufacturing process on compound stability and to confirm the biological activity of the developed suppositories [39].

### 3.3. In Vitro Diffusion Study

Based on the results of the release of active substances from the suppositories, the following were selected: hydrophobic and hydrophilic bases for the production of chamazulene-containing suppositories. The suppository comprising a base of cocoa butter, vegetable oil, Tween 80, and CO_2_ extract of *R. officinalis* L. herb proved to be the most optimal in terms of chamazulene release.

The release kinetics of chamazulene from different suppository bases were compared. The observed differences in release profiles may be attributed to the lipophilicity of the active compounds and the physicochemical properties of the base.

The present agar diffusion results do not provide compound-specific evidence of chamazulene release. The literature does not establish rosemary extract as a primary source of chamazulene, and studies specifically examining chamazulene release from rosemary-based formulations are limited [40,41]. Therefore, the observed colored zones should be interpreted only as evidence of the diffusion of colored extract constituents rather than as confirmed chamazulene release. Further investigation should include gas chromatography–mass spectrometry (GC–MS) analysis of both the initial CO_2_ extract and the acceptor medium. GC–MS is appropriate for confirming chamazulene and other volatile or semivolatile terpenoid constituents through their mass spectra and retention indices. If chamazulene is replaced with a nonvolatile rosemary marker, high-performance liquid chromatography (HPLC) would be more suitable for the determination of diterpenes such as carnosol and carnosic acid. Compound-specific measurements in the acceptor medium at predetermined time points would be required to characterize release quantitatively.

### 3.4. General Discussion and Limitations

Taken together, the results of this study demonstrate that the quality of suppositories containing the CO_2_ extract of *R. officinalis* L. herb depends on a combination of formulation and process parameters. The optimized preparation temperature (70 °C), stirrer speed (120 rpm), and cooling regime (8 °C, 60 min) ensure the technological stability, uniformity, and reproducibility of the suppository mass.

At the same time, several limitations should be acknowledged:

1. Chamazulene as a marker: The choice of chamazulene as a quality-control marker for a rosemary-based formulation requires additional justification, since this compound is not a characteristic major constituent of rosemary extract. Alternative markers, such as carnosic acid, carnosol, or 1,8-cineole, may be more appropriate and should be considered in future studies.
2. In vitro release model: The equilibrium dialysis method provides useful comparative data but does not fully reproduce the physiological conditions of the rectum. Further studies using biorelevant media and ex vivo models are warranted.
3. Analytical confirmation: The identity of chamazulene in the test samples was confirmed by retention time and spiking. Additional confirmation by mass spectrometry (GC–MS) or nuclear magnetic resonance would strengthen the analytical validity of the results.
4. Biological activity: The pharmacological efficacy of the developed suppositories was not evaluated in this study. Further in vivo studies are required to confirm the anti-inflammatory and antioxidant effects of the formulation.

## 4. Materials and Methods

### 4.1. Plant Material and Preparation of the CO_2_ Extract

The above-ground part of *R. officinalis* L. was collected in July 2024 in the Turkestan Region of the Republic of Kazakhstan. The CO_2_ extract was prepared from the plant material using a UE-1 carbon dioxide extraction unit at a temperature of 50 °C and a pressure of 200 bar [4].

### 4.2. Preparation of Suppositories Containing a CO_2_ Extract of Rosmarinus officinalis L.

The nominal weight of each suppository was 1.5 g. Each suppository contained 0.5 g of a CO_2_ extract of *R. officinalis* L., 0.6 g of cocoa butter, 0.1 g of yellow beeswax, 0.25 g of linseed oil, and 0.05 g of Tween-80. 96% ethanol was used as an excipient in the preparation of the concentrate.

To prepare the concentrate, the CO_2_ extract was mixed with 96% ethanol and a portion of the flaxseed oil. Cocoa butter and yellow beeswax were melted at 70 °C, after which half of the flaxseed oil was added to the molten base. The prepared extract concentrate, the remaining half of the flaxseed oil, and Tween 80 were then gradually added under continuous stirring. The suppository mass was stirred until a homogeneous consistency was achieved, poured into molds, and cooled at 8 °C for 60 min. After cooling, the suppositories were removed from the molds, visually inspected, and packaged.

Visual inspection was used only for a preliminary assessment of the absence of visible separation and macroscopic heterogeneity in the suppository mass. The uniformity of chamazulene distribution was quantitatively assessed in samples selected from different units of the manufactured batch (n = 5). The results are presented as the mean ± standard deviation (SD).

Given that the CO_2_ extract of *R. officinalis* L. accounts for 0.5 g of the suppository’s mass, it is advisable to prepare a concentrate in advance by dissolving the active ingredients in ethyl alcohol and a portion of vegetable oil.

The uniformity of distribution of the CO_2_ extract of *R. officinalis* L. was determined as follows: Every 5 min, spot samples of 0.4 mL were taken with a pipette from the lower and upper parts of the reactor; these were quantitatively transferred to a microscope slide and visually assessed for uniformity of the mass. The results showed that temperature has little effect on the uniformity of distribution in the concentrate. The determining factor in this case is the stirrer speed. The optimal operating condition is considered to be a speed of 60 rpm, which allows the system to become homogeneous within 25 min even at a temperature of 37 °C. This temperature ensures the required mixture structure and is more favorable for easily oxidizable compounds. Increasing the rotation speed is not economically feasible. The study parameters are presented in Table 4.

### 4.3. Determination of Mechanical Strength

To select the optimal molding conditions for suppositories, the effect of temperature and cooling duration on the mechanical strength of the finished samples was investigated. After being poured into molds, the suppository mass was cooled under the temperature–time conditions under investigation. For each condition, five samples (n = 5) were examined. The results are presented as the mean ± standard deviation (SD).

### 4.4. Quantitative Determination of Chamazulene in Suppositories

The quantitative content of chamazulene in suppositories containing a CO_2_ extract of *R. officinalis* L. was determined by gas chromatography with flame ionization detection (GC–FID) on a Cristallux 4000M gas chromatograph (Meta-Chrome, Yoshkar-Ola, Russia).

Approximately 1.5 g of the suppository preparation, accurately weighed, was dissolved in 15 mL of chloroform under gentle heating. The resulting solution was quantitatively transferred to a 25 mL volumetric flask, made up to the mark with chloroform, and mixed thoroughly. An aliquot of 1.0 µL of the prepared solution was injected into the chromatograph using a microsyringe. The chromatograms of the hamazulene standard and the test suppository sample are shown in Figure 2 and Figure 3, respectively.

Chromatographic conditions: flame ionization detector (FID). A 30 m long ZB-5 capillary column (a copolymer of 5% phenyl and 95% dimethylpolysiloxane) with an internal diameter of 0.25 mm and a film thickness of 0.25 µm. Carrier gas: argon at a flow rate of 20 cm^3^/min; hydrogen—60 cm^3^/min; air—550 cm^3^/min. Initial column temperature: 35 °C, rising to 270 °C at a rate of 2 °C per minute. Detector temperature: 270 °C. Evaporator temperature: 250 °C. Each sample was chromatographed at least five times.

Simultaneously, the chamazulene standard solution (SS) was analyzed; 1.0 µL of the solution was injected into the chromatograph evaporator using a microsyringe at least five times.

The chamazulene content of the preparation (X), expressed as a percentage, was determined using the external standard method according to the following formula:

(1) $$X=\frac{S_{1}\cdot m_{0}\cdot 25\cdot 100\cdot P}{S_{0}\cdot m_{1}\cdot 25\cdot 100}$$

where S_1_ is the mean of the peak areas of chamazulene on the chromatograms of the test solution;

S_0_ is the mean of the peak areas of chamazulene on the chromatograms of the chamazulene standard solution;

m_1_ is the mass of the test sample, in grams;

m_0_ is the mass of the chamazulene standard solution, in grams;

P is the quantitative content of chamazulene in the chamazulene standard solution, in grams;

25 is the dilution factor.

Preparation of a standard solution of chamazulene.

An accurately weighed 0.5 g portion of the CO_2_ extract of *R. officinalis* L. was transferred to a 25 mL volumetric flask and dissolved in 15 mL of chloroform. The solution was diluted to volume with the same solvent and mixed thoroughly. A freshly prepared solution was used.

### 4.5. Biopharmaceutical Study of Suppositories

To investigate the release of chamazulene, agar gel was poured into Petri dishes in 10 mL portions. Once the agar had set, wells were made on its surface using an 8 mm diameter stainless steel tube. Melted samples of the suppository compositions, weighing 0.1 g each, were placed into the wells. The dishes were covered and incubated at (37 ± 0.5) °C for 24 h. After 24 h, the diameter of the colored zones around each test sample was measured using a Vernier caliper and expressed in millimeters. The agar gel diffusion method was used for a comparative assessment of the spread of the colored components of the extract from the suppository compositions under in vitro conditions. The diameter of the stained zone served as an indirect indicator of diffusion.

The release of the active substance into the agar gel was assessed visually. The yellow-green coloration of the zone around the sample was preliminarily attributed to the spread of the stained components of the extract and the suppository base into the agar gel.

Each formulation was tested in five replicates (n = 5), with the results expressed as the mean ± standard deviation (SD).

## 5. Conclusions

This preliminary study identified key manufacturing parameters for the previously developed suppository formulation containing a CO_2_ extract of *R. officinalis* L.: preparation of the base at 70 °C and 120 rpm for 12 min, preparation of the extract concentrate at 37 °C and 60 rpm for 25 min, mixing of the suppository mass at 60 rpm for 20 min, and cooling at 8 °C for 60 min. The principal technological advantage was the reduction in the mixing time from 100 to 20 min through preliminary preparation of the extract concentrate. In contrast to our previous study [4], which focused on formulation selection, physicochemical characterization, and pharmacological activity without defining the manufacturing parameters, the present work provides an initial experimentally based framework for process optimization. Nevertheless, the findings should be regarded as preliminary because the identity of the proposed marker has not yet been conclusively confirmed, the GC–FID method has not been fully validated, quantitative content uniformity and stability have not been established, and the agar diffusion assay does not provide compound-specific release or bioavailability data. Further studies should therefore include confirmation of the marker by gas chromatography–mass spectrometry (GC–MS) using mass-spectral and retention-index data, full validation of the quantitative method, assessment of content uniformity in individual suppositories, stability studies under defined storage conditions, and biorelevant release testing with compound-specific quantification in the acceptor medium.

## Figures and Tables

**Table 1:** Composition of suppositories containing *Rosmarinus officinalis* L. CO_2_ extract [4].

Component	Amount per Suppository
CO_2_ extract of *R. officinalis* L. herb	0.50 g
Cocoa butter (Olvea Group, France; Ph. Eur.)	0.60 g
Polysorbate 80 (Tween 80) (Acros Organics, Belgium)	0.05 g
Flaxseed oil (Prombaza-7 LLP, Kazakhstan; Ph. Eur.)	0.25 g
Yellow beeswax (KahlWax, Germany, Ph. Eur.)	0.10 g
Ethanol (Dosfarm, Kazakhstan; pharma grade))	q.s.
Nominal suppository mass	1.50 g

Note: q.s.—quantity sufficient. The nominal mass of 1.50 g represents the sum of the listed components excluding ethanol.

**Table 2:** Duration of base preparation depending on temperature and mixer speed (n = 5 independent experiments per condition).

No.	Stirrer Speed, rpm	Mixing Time, min		
		60 °C	70 °C	80 °C
1	30 rpm	>180	120	60
2	60 rpm	>180	35	25
3	120 rpm	150	12	10
4	150 rpm	120	12	9

Note: Values are presented as mean ± standard deviation (SD).

**Table 3:** Mixing time of the suppository mass depending on temperature, stirrer speed, and prior preparation of the concentrate (n = 5).

Stirrer Speed, rpm	Mixing Time, min		
	37 °C	45 °C	60 °C
With concentrate preparation			
30 rpm	45	40	40
60 rpm	20	20	20
120 rpm	15	15	15
Without concentrate preparation			
30 rpm	140	120	110
60 rpm	100	100	100
120 rpm	90	90	80

**Table 4:** Process parameters used during the stages of preparing the suppository mass.

Parameter	Values	Stage/Application	Purpose
Temperature	60/70/80 °C	Base preparation	Selection of the temperature for wax dissolution
Temperature	37/45/60 °C	Suppository mass preparation, with and without prior concentrate preparation	Comparison of homogenization rates
Stirrer speed	30/60/120/150 rpm	Base preparation; suppository mass preparation	Assessment of the effect of stirring on processing time
Stirrer speed	30/60/120 rpm	Method without prior concentrate preparation	Evaluation of the alternative preparation method
Time	Until dissolution/until homogeneity	All stages	Primary recorded outcome
Number of replicates (n)	5	Suppository mass preparation	Assessment of experimental repeatability

## Data Availability

The original contributions presented in this study are included in the article. Further inquiries can be directed to the corresponding authors.
